# A Review of Limbic System-Associated Membrane Protein in Tumorigenesis

**DOI:** 10.3390/biomedicines12112590

**Published:** 2024-11-13

**Authors:** Kayleigh Wittmann Sinopole, Kevin Babcock, Albert Dobi, Gyorgy Petrovics

**Affiliations:** 1 School of Medicine, Uniformed Services University of the Health Sciences, Bethesda, MD 20814, USA; kayleigh.sinopole@usuhs.edu; 2 Center for Prostate Disease Research, Murtha Cancer Center Research Program, Department of Surgery, Uniformed Services University of the Health Sciences, Bethesda, MD 20817, USA; babcock_kevin@yahoo.com (K.B.); dobi.albert@gmail.com (A.D.); 3 Walter Reed National Military Medical Center, Bethesda, MD 20889, USA; 4 Henry M. Jackson Foundation for the Advancement of Military Medicine, Bethesda, MD 20817, USA

**Keywords:** limbic system-associated membrane protein (LSAMP), epithelial–mesenchymal transition (EMT), cell adhesion, RTK pathway

## Abstract

Purpose of Review: This review aims to describe the role of limbic system-associated membrane protein (LSAMP) in normal- and pathophysiology, and its potential implications in oncogenesis. We have summarized research articles reporting the role of LSAMP in the development of a variety of malignancies, such as clear cell renal cell carcinoma, prostatic adenocarcinoma, lung adenocarcinoma, osteosarcoma, neuroblastoma, acute myeloid leukemia, and epithelial ovarian cancer. We also examine the current understanding of how defects in LSAMP gene function may contribute to oncogenesis. Finally, this review discusses the implications of future LSAMP research and clinical applications. Recent Findings: LSAMP has been originally described as a surface adhesion glycoprotein expressed on cortical and subcortical neuronal somas and dendrites during the development of the limbic system. It is categorized as part of the IgLON immunoglobulin superfamily of cell-adhesion molecules and is involved in regulating neurite outgrowth and neural synapse generation. LSAMP is both aberrantly expressed and implicated in the development of neuropsychiatric disorders due to its role in the formation of specific neuronal connections within the brain. Additionally, LSAMP has been shown to support brain plasticity via the formation of neuronal synapses and is involved in modulating the hypothalamus in anxiogenic environments. In murine studies, the loss of LSAMP expression was associated with decreased sensitivity to amphetamine, increased sensitivity to benzodiazepines, increased hyperactivity in new environments, abnormal social behavior, decreased aggressive behavior, and decreased anxiety. Findings have suggested that LSAMP plays a role in attuning serotonergic activity as well as GABA activity. Given its importance to limbic system development, LSAMP has also been studied in the context of suicide. In malignancies, LSAMP may play a significant role as a putative tumor suppressor, the loss of which leads to more aggressive phenotypes and mortality from metastatic disease. Loss of the LSAMP gene facilitates epithelial-mesenchymal transition, or EMT, where epithelial cells lose adhesion and gain the motile properties associated with mesenchymal cells. Additionally, LSAMP and the function of the RTK pathway have been implicated in tumorigenesis through the modulation of RTK expression in cell membranes and the activation of second messenger pathways and β-catenin. Summary: Beyond its many roles in the limbic system, LSAMP functions as a putative tumor suppressor protein. Loss of the LSAMP gene is thought to facilitate epithelial-mesenchymal transition, or EMT, where cells lose adhesion and migrate to distant organs. LSAMP’s role in modulating RTK activity and downstream ERK and Akt pathways adds to a large body of data investigating RTK expression in oncogenesis. The characteristics of LSAMP defects and their association with aggressive and metastatic disease are evident in reports on clear cell renal cell carcinoma, prostatic adenocarcinoma, lung adenocarcinoma, osteosarcoma, neuroblastoma, acute myeloid leukemia, and epithelial ovarian cancer.

## 1. Introduction

Prostate cancer is a common malignancy and one of the leading causes of cancer deaths [1]. Prostate cancer diagnosis rates and diagnoses with advanced disease are increasing. Prostate cancer mortality among patients of African ancestry is 2- to 4-times higher than in patients of other ancestries [2]. The mortality of prostate cancer is largely due to inherited factors, and ancestry-associated germline mutations have been found to lead to more aggressive cancer and early disease progression [3]. In somatic contexts, genomic structural variations have been linked to the development of metastatic castration-resistant prostate cancer [4]. A growing body of evidence points to how the LSAMP gene functions in prostate cancer, as well as other cancers, and the role of LSAMP in tumor suppression.

## 2. History of LSAMP Research

Limbic system-associated membrane protein (LSAMP) is a surface glycoprotein expressed on cortical and subcortical neuronal somas and dendrites during the development of the limbic system [5]. LSAMP is categorized as part of the immunoglobulin-like family of cell-adhesion molecules IgLON, which also includes opioid-binding cell adhesion molecule OBCAM, neurotrimin, neuronal growth regulator 1, and IgLON5 [6]. LSAMP is involved with other members of the IgLON family in regulating neurite outgrowth and neural synapse generation [7]. All IgLON members are found at neuronal synapses, where they form homophilic and heterophilic dimers [8]. They can interact on the same membrane in a *cis* configuration, or span the membrane and synaptic cleft in a trans configuration [8,9]. In addition to expression in neural tissue, IgLONs are expressed in tissues outside the nervous system, including in the heart, lung, liver, small intestine, adrenal glands, kidney, male ureter, vas deferens, ovary, and uterus [9]. LSAMP expression specifically is measurable across the genitourinary system, including in the kidney, ureter, testis, and vas deferens [9].

Structurally, all members of the IgLON family comprise three immunoglobulins connected together and attached to the cell membrane by the glycolipid glycosylphosphatidylinositol [8]. Despite their homology in structure, IgLONs have not demonstrated redundancy in function, and when an IgLON family member is deficient, no increase in another IgLON protein is seen [9]. From their attachment points on neuronal surfaces, IgLONs interact with other IgLONs in the synaptic gap, connecting via surfaces made up of beta strands [8]. Neurotrimin (NTM) is known to interact with neuronal growth regulator 1 (NEGR1) [8]. Both proteins comprise three immunoglobulins in series connected by beta strands, where the Ig1 surfaces superimpose on each other to form IgLON dimers [8]. Similarly, LSAMP and NTM have been shown to have strong affinity in both trans and cis configurations [10]. Based on studies of LSAMP knockout, NTM knockout, and double knockout mice, it has been proposed that NTM might serve as a negative regulator of early neurite outgrowth while LSAMP enhances outgrowth, as a loss of NTM results in premature neurite development but only in the presence of LSAMP [10]. In hippocampal cultures, NTM was shown to induce tissue proliferation but only in the presence of LSAMP [10]. In neuronal cultures, LSAMP appeared to increase apoptosis but only in the presence of NTM [10]. The relationship between LSAMP and NTM emphasizes the influence of IgLON family interactions in both physiologic and pathologic states.

The function of LSAMP independent from interactions with other IgLONs has been investigated in studies spanning neuroscience and behavioral science. LSAMP is aberrantly expressed in and is implicated in the development of neuropsychiatric disorders, especially schizophrenia, due to its normal function forming specific neuronal connections within the brain [7]. Additionally, LSAMP has been shown to support brain plasticity via the formation of neuronal synapses, especially in the hippocampus, and is involved in modulating the hypothalamus in stressful or novel environments [5]. Studies in mice with wild-type, heterozygous, and homozygous loss of LSAMP expression have demonstrated that LSAMP loss results in decreased sensitivity to amphetamine with increased serotonin release and subsequently blunted serotonin turnover, as well as decreased serotonin at baseline compared with wild-type mice [11]. The loss of LSAMP in mice also resulted in hyperactivity in new environments, less aggressive behavior, less anxiety, and less whisker trimming, a marker of social dominance [7,11]. The loss of LSAMP in mice increased the anxiolytic and hypnotic effects of benzodiazepines [7]. These findings suggest that LSAMP modulates both serotonergic activity via presynaptic serotonergic neurons in the brainstem, and GABA activity via the modulation of GABAA receptor subunits [7,11]. Lastly, LSAMP alterations have an association with suicide, which was investigated due to genomic structural differences in the limbic system among suicide victims [12]. Thus, the function of LSAMP as a cell-adhesion protein is important to understanding neural development in the limbic system [12,13]. Beyond its many roles in the limbic system, LSAMP has been suggested to function as a tumor suppressor in early studies [14,15,16].

In malignancies, LSAMP may play a significant role as a tumor suppressor, the loss of which leads to more aggressive phenotypes and mortality from metastatic disease. The loss of LSAMP precipitates epithelial–mesenchymal transition (EMT), where epithelial cells lose adhesion and gain the motile properties associated with mesenchymal cells [6]. This transition rids cells of apical–basal polarity and allows them to break down the extracellular matrix, lose adhesion, and migrate away from the basement membrane and into vasculature where they continue their migration [17]. The process of EMT permits the formation of new structures in embryonal development, but also allows for metastasis when it occurs in the setting of oncogenesis [6]. The EMT that occurs in oncogenesis displays some differences from normal physiologic EMT, including in the EMT markers that cancer cells display, and the cells themselves can be epithelial-like, less mesenchymal, often with an incomplete transition [17]. The process of EMT in oncogenesis may imply that only a few cells, having reverted to a mesenchymal stem cell-like state, are required for tumor metastasis and invasion in a distant organ, consistent with the cancer stem-cell phenotype [6]. These characteristics of LSAMP defects are evident in reports on clear cell renal cell carcinoma, lung adenocarcinoma, prostatic adenocarcinoma, osteosarcoma, neuroblastoma, acute myeloid leukemia, and epithelial ovarian cancer (Table 1).

LSAMP defects have been implicated in multiple cancers. These malignancies have poor outcomes affecting large numbers of patients. For example, renal cell carcinoma is typically discovered incidentally on imaging and incidence increases yearly, with over 81,610 new diagnoses and 14,390 deaths estimated for 2024 [1]. Familial RCC occurring due to inherited genetic mutations encompasses 2–8% of tumors, but up to 16% of advanced RCC demonstrates mutations in cancer-associated genes [25]. Lung cancer remains the leading cause of cancer death in the U.S. across genders, and while incidence is declining annually, only 1 in 4 patients will survive to five years [1]. Osteosarcoma is an aggressive cancer and the most common primary malignancy of bone, with a 65–66% 5-year survival in patients younger than 20 years [1]. Brain cancer is the leading cause of cancer death in children younger than 20 years, and 18% of patients with neuroblastoma will die within five years [1]. Acute myeloid leukemia will see an estimated 20,800 new cases and 11,220 deaths in 2024, and is the second most common leukemia in adults and children, with mortality and risk of invasive disease increasing with age [1]. Ovarian cancer is the leading cause of death from gynecologic cancer, where approximately 50% of patients present with disease that has already metastasized, and less than half of patients survive to five years [26]. Given the heavy mortality load of cancers of every type, identifying the specific factors causing tumor aggression and decreased survival is crucial.

## 3. Renal Cell Carcinoma

Renal cell carcinoma comprises most primary renal neoplasms and develops sporadically from tubular epithelium in the renal cortex. RCC can arise in a nonhereditary fashion or can occur as part of an inherited syndrome, such as Von Hippel–Lindau syndrome, Birt–Hogge–Dube syndrome, and tuberous sclerosis. Clear cell is the most common subtype of RCC and develops from the proximal renal tubule along with papillary RCC, while chromophobe RCC develops from the collecting tubule [27].

The inheritance of clear cell renal cell carcinoma (CCRCC) is primarily due to mutations in VHL, but chromosomal translocations on chromosome 3 have also been identified in both VHL-related RCC and non-VHL hereditary RCC [18,27]. However, many of the hereditary forms of RCC which display a translocation and loss of the derivate chromosome 3 allele do not have VHL mutations [18]. The translocations implicated in the development of these hereditary forms of RCC span multiple genes, all of which are located on chromosome 3 [18]. The literature reports eight chromosomal translocation families linked to chromosome 3, suggesting that these translocation families might contribute to the development of CCRCC in ways similar to VHL [18].

One of the genes located on chromosome 3 involved in a translocation implicated in hereditary CCRCC, is LSAMP [18]. The LSAMP gene located at 3q13.3 has been identified as part of a translocation t(1;3) (q32.1;q13.3) associated with the development of CCRCC [18]. In an analysis of nine CCRCC cell lines, 53 sporadic tumors, and four familial tumors, no mutations in LSAMP itself were discovered, but the downregulation of LSAMP expression was observed in nearly all the tumor samples [18]. Epigenetic silencing due to LSAMP promoter methylation was discovered in 78% of CCRCC cell lines, 26% of sporadic tumors, and 100% of familial tumors, and the expression of LSAMP increased following the application of DNA methyl transferase inhibitors [18]. Additionally, the loss of heterozygosity of the LSAMP locus was found to be significantly associated with tumor development and occurred independently of LSAMP promotor methylation [18].

In RCC cell line models lacking LSAMP expression, activations or re-expression of LSAMP resulted in the suppression of tumor growth. Plasmids expressing green fluorescent protein EGFP-LSAMP were introduced to two RCC cell lines containing methylated LSAMP promotors, and these cells were allowed to proliferate along with control cells expressing green fluorescent protein EGFP alone. While cells expressing EGFP alone grew like non-transfected cells, EGFP-LSAMP-transfected cells showed a significant reduction in cell growth, and these cells did not undergo apoptosis [18].

## 4. Prostatic Adenocarcinoma

The disparity in prostate cancer mortality between American men of African Ancestry (AA) and those of Caucasian Ancestry (CA) has been highlighted in numerous studies. Although to a lesser extent, prostate cancer disparities are also apparent in equal-access healthcare settings, arguing for the examination of biologic and genetic factors. While the presence of commonly observed ERG and PTEN genomic defects in prostate tumor genomes has been highlighted in previous studies, these studies were conducted primarily on datasets of tumors from patients of CA [3]. The inactivation of LSAMP has been understood as a cancer genomic event via deletion involving the LSAMP locus at chromosome 3q13.31, which occurs at significantly higher rates in tumors of patients of AA than CA [3]. Deletions involving the LSAMP locus were reported in 27% of AA compared with 13% of CA tumors in tumor copy number variation data, and in 26% of AA compared with 7% of CA patients by Fluorescence in situ Hybridization Assays (FISH) on tumor tissue microarrays [3]. Further, the association of LSAMP deletion with rapid disease progression suggests that detecting LSAMP deletions may serve as a predictor of disease progression in patients of AA [3]. An analysis of structural variations in 180 prostate cancer tumors identified LSAMP as a candidate driver gene and possible prognostic target for AA tumors [28]. The chromosomal mapping of prostate tumor DNA breakpoints in a recent report pinpointed the LSAMP locus and its association with localized and metastatic castration-resistant prostate cancers [4].

**Figure 1:**
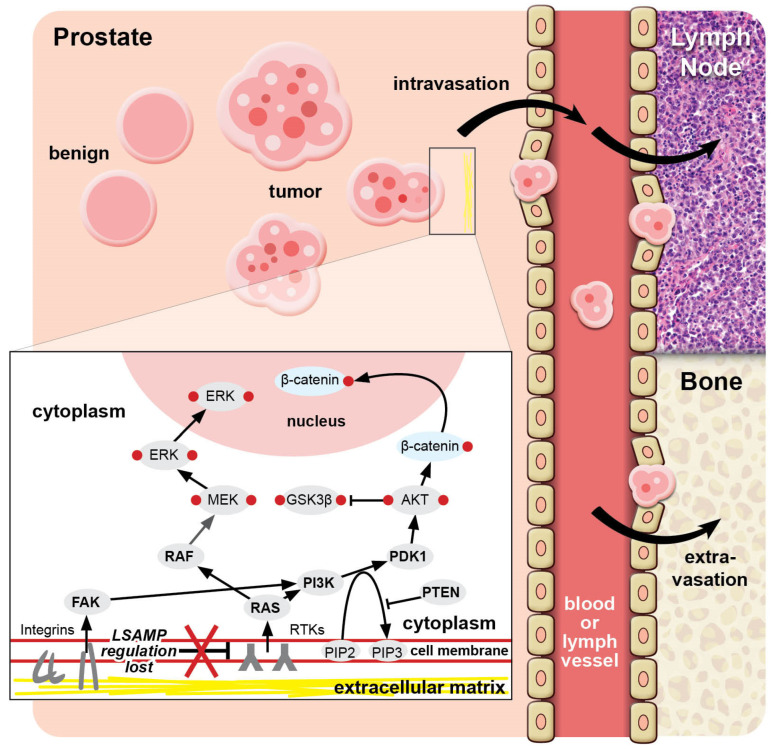
LSAMP in prostate cancer. The loss of LSAMP in the cell membrane upregulates RTK signaling and downstream ERK and Akt pathways. Deletion in LSAMP results in the loss of adhesion to normal extracellular matrix elements, leading to detachment of a nest of tumor cells from the primary tumor site and attachment to lymphatic or vascular endothelium where the LSAMP-deleted cancer cells spread to distant sites, such as lymph node and bone. Abbreviations: ERK, extracellular signal-regulated kinase; FAK, focal adhesion kinase; MEK, mitogen-activated protein kinase; RTK, receptor tyrosine kinase.

The LSAMP homologue, OPCML, a tumor suppressor and cell adhesion protein and member of the IgLON family, regulates downstream receptor tyrosine kinases affecting the extracellular signal-regulated kinase (ERK), as well as β-catenin through the Akt signaling pathway [29]. In prostate cancer cell culture model experiments, the absence of LSAMP affected RTKs and activated both the ERK and Akt pathways and downstream β-catenin through RAS signaling [23]. This is one of the canonical pathways in receptor tyrosine kinase to RAS-to-RAF activity that affects cell adhesion in the cancer context [30]. Parallel to RAS activation, Akt can be activated by focal adhesion kinase, FAK, in response to integrin signals [31,32]. Taken together, LSAMP deletion and the subsequent alteration of integrins may promote the loss of adhesion of the cell to the basement membrane in known pro-metastatic pathways associated with disease progression (Figure 1). Thus, the normal expression of LSAMP may act as a stabilizer of cell-to-basement membrane adhesion. Additionally, altered cellular metabolism as evidenced in metabolomic data, and emerging deutenomic data, plays a role in tumorigenesis [33]. Increases in β-catenin associated with activated Wnt signaling have been shown to lead to the metabolic changes seen in cancer contexts [33]. The regulation of fatty acid metabolism by β-catenin has been investigated in studies of prostate cancer. In the CAPS2 trial, patients with biochemical recurrence of prostate cancer after definitive treatment followed a low-carbohydrate diet for six months, and researchers found that high serum levels of TCA metabolites associated with ketogenesis, such as 2-hydroxybutyric acid and ketone bodies, correlated with longer PSA doubling time [34].

## 5. Lung Adenocarcinoma

Lung cancer remains the primary cause of cancer death worldwide, and lung cancer mortality is marked by aggressive metastasis, which occurs due to the EMT mechanism and subsequent mesenchymal–epithelial transition, or MET, once the cancer cells reach their new organ [17]. LSAMP alterations have been reported in the context of lung cancer progression [6]. In an LSAMP gene expression study, a small set of lung adenocarcinoma samples was compared with non-malignant control samples, suggesting that LSAMP was expressed at lower levels in the cancer tissue [6]. LSAMP, more so than any other member of the IgLON family, was associated with cancer aggressiveness, where lower levels of LSAMP expression resulted in increased nodal metastasis and more advanced tumor stage [6]. Survival analyses associated decreased expression of LSAMP with shorter survival time [6]. Other members of the IgLON family did not significantly affect survival in this study. Hypermethylation, copy number variation, and microRNA post-translational mechanisms were also investigated as possible regulatory mechanisms of LSAMP expression in lung adenocarcinoma. Copy number variation and microRNA appeared to be unlikely contributors to LSAMP regulation, but the methylation of the LSAMP promotor resulted in decreased copies of LSAMP mRNA, increased nodal metastasis, and more advanced tumor stages [6]. The role of LSAMP in the epithelial–mesenchymal transition EMT was also investigated. In cell culture models, markers of EMT were found to be modified in response to the silencing of LSAMP via short hairpin RNA [6]. The expression of epithelial marker E-cadherin decreased, while mesenchymal markers such as N-cadherin, vimentin, alpha-smooth muscle actin, and EMT-associated zinc finger proteins Snail and Slug, increased [6].

## 6. Osteosarcoma

Osteosarcoma is an aggressive malignancy and a common primary bone tumor. Osteosarcomas are most frequently found in children, with a smaller spike seen in ages over 50 years, and they are associated with poor prognosis due to their high grade at diagnosis and frequent metastases [14]. Osteosarcomas develop at sites of bone growth, typically at the metaphysis, and most commonly around the knee at the proximal tibia, proximal humerus, or distal femur [14].

LSAMP has been investigated for its tumor-suppressive role in osteosarcoma and was reported in several studies. A dataset of 36 samples of human osteosarcoma tumors and 20 osteosarcoma cell lines was analyzed for DNA copy number changes, from which gains or losses of chromosomal regions were assessed [14]. The chromosomal locus 3q.13.31, harboring LSAMP, had a deletion rate of 56% in the tumor samples [14]. Another study on a dataset of 49 tumor biopsy tissues and DNA samples also identified locus 3q13.31 as the most common deletion in osteosarcoma, and an analysis of 43 tumor biopsies with the addition of 5 osteosarcoma cells lines demonstrated decreased LSAMP expression in over 64% of cases [15]. A subsequent study further investigating LSAMP on chromosomal region 3q13.31 found reduced copy number of LSAMP in 59% of osteosarcoma samples and reduced expression of LSAMP in 60% of samples [21]. An analysis of a database of single-cell osteosarcoma messenger RNA expression data confirmed aberrant transcription levels in osteoblasts and in carcinoma-associated fibroblasts, significantly more than in other cell types [35]. The methylation of the LSAMP promoter region was suggested as the epigenetic mechanism contributing to the decreased expression of LSAMP in osteosarcoma, as in RCC and lung adenocarcinoma. From a total number of tumor samples and cell lines that showed either the partial or full methylation of the LSAMP promoter, 60% had a subsequent decreased expression of LSAMP [14].

LSAMP expression has also been shown to affect tumor growth and proliferation in osteosarcoma. Decreased expression of LSAMP via small interfering RNA resulted in the enhanced proliferation of osteoblast cells compared with controls [15]. When comparing cell lines with and without LSAMP expression, the presence of LSAMP was significantly associated with a 15–20% reduction in subsequent cell growth [21]. Further, LSAMP expression in mouse osteosarcoma models resulted in significant delay in tumorigenesis. In these osteosarcoma models, survival analyses linked decreased LSAMP mRNA expression with worsened survival [14]. Mechanistically, the suppression of LSAMP by siRNA increased the transcription of cyclin A2 and cyclin B1, pointing to the role of LSAMP in affecting the cell cycle [15].

## 7. Neuroblastoma

Neuroblastoma is a primarily pediatric neuroendocrine malignancy that develops from neural crest precursor cells. Structural alterations in LSAMP at chromosomal region 3q13.31 have been found in whole genome sequencing studies of relapsed and high-risk neuroblastoma [24]. Heterozygous rearrangements in 3q13.31 at the region of LSAMP were present in 17% of tumor samples, while 43% of neuroblastoma cell lines displayed segmental chromosomal alterations in LSAMP [24]. When LSAMP expression was silenced via short hairpin RNA, significantly increased cell growth was noted in neuroblastoma cell lines [24]. Following transfection with LSAMP in cell lines with heterozygous and homozygous LSAMP deletions, a significant decrease in growth was observed [24]. For a dataset of 498 neuroblastoma tumors, decreased LSAMP expression was significantly associated with decreased event-free survival and overall survival [24]. In neuroblastoma, LSAMP, as in RCC and osteosarcoma, appears to function as a tumor suppressor correlated with increased survival.

## 8. Acute Myeloid Leukemia

LSAMP defects have been identified in acute myeloid leukemia (AML), specifically in the cytogenetic category of AML associated with either t(8;21) or inv(16), referred to as core-binding factor-AML, or CBF-AML [20]. CBF-AML has a good prognosis in both adult and pediatric populations, but most adult patients, and up to 20% of pediatric patients, will eventually experience relapse [20]. Of 39 bone marrow samples of CBF-AML that relapsed, matched with samples taken at diagnosis, the deletion of chromosomal region 3q13.31 that includes LSAMP was the most common copy number alteration, occurring in 12% [20]. The larger deletions associated with relapses were all shown to include the deletion of LSAMP, and the minimally deleted region associated with relapse samples was found to contain exons of LSAMP [20].

## 9. Ovarian Epithelial Carcinoma

Ovarian epithelial cancer is the most common type of ovarian cancer and is a leading cause of ovarian cancer mortality [19]. It is not well understood where ovarian epithelial cancer originates, but an increasing number of reports suggest it originates in the epithelial lining of the uterine tubes [36,37]. Physiologically normal ovarian epithelium is a simple squamous to simple cuboidal epithelium, but unlike true epithelial tissue, exhibits attributes of mesenchymal cells [19,38]. Ovarian epithelium displays a more fragile adherence to the basement membrane than in typical epithelium, minimal expression of E-cadherin except in metaplasia and other instances where the epithelium becomes more columnar, and characteristics that support repair of the ovarian surface following follicular rupture, including contractile capacity, motility, and proliferative capability [38]. These attributes give ovarian epithelium similarities to the process of EMT underlying the role of LSAMP defects in tumorigenesis and indicate that ovarian epithelium is not well-differentiated and prone to malignant transformation [19,38].

LSAMP has been linked to ovarian cancer, similar to the IgLON family member OBCAM, where the pathophysiology of the disease is better understood. To some extent, LSAMP expression was found to be reduced in all types of epithelial ovarian cancers, but to a statistically significant level it was linked to the endometrioid subtype [19]. An analysis of cancer genomic copy number variations in serous ovarian cancer identified focal losses of LSAMP in malignant, but not in normal tissues [22]. In studies on OBCAM, the presence of OBCAM was shown to have a tumor suppressive effect via extracellular binding to receptor tyrosine kinases and the subsequent attenuation of downstream pathways, specifically, inhibition of the phosphorylation of ERK1, ERK2, and Akt [39]. The introduction of OBCAM in ovarian cancer cell culture models resulted in significantly decreased growth [39]. In mice, intraperitoneal injections of OBCAM-expressing malignant cells resulted in significantly decreased rate of tumorigenesis, decreased development of ascites, and decreased peritoneal extension of ovarian cancer cell lines, compared with controls [39]. These data are consistent with findings showing that in prostate cancer cells, the modulation of the receptor tyrosine kinase pathways is a potential mechanism by which LSAMP enacts its tumor suppressive activity [23].

Interestingly, high levels of LSAMP expression were seen in moderately differentiated and well-differentiated ovarian cancers, but were associated with decreased overall survival. Thus, measuring the levels of LSAMP expression may help prognosing a poor outcome beyond the morphological differentiation status [19]. The reason for this is not well understood at this time, but it is possibly related to the role of LSAMP in the physiologically normal ovary, and the fact that normal ovarian epithelium is not well-differentiated compared to other epithelial tissues.

## 10. Discussion

LSAMP is a cell-surface adhesion molecule originally known for its role in the limbic system and in the development of neuropsychiatric disorders. LSAMP was shown to have tumor suppressive activity in a variety of cancers, first in renal cell carcinoma, where the LSAMP gene was found to be collocated with VHL on chromosome 3, with decreased LSAMP expression observed in nearly all the tumor samples and cell lines of clear cell RCC [18,27]. In RCC, LSAMP was shown to suppress growth in vitro and this suppression was reversed with a DNA methylation inhibitor [18]. Studies of ovarian epithelial carcinoma reported that LSAMP was reduced in tumor samples and was a prognostic factor, associated with decreased survival [19]. Osteosarcoma studies also linked LSAMP to worsened survival and found that a heterologous expression of LSAMP delayed tumor growth in vivo [21]. In neuroblastoma, LSAMP suppressed growth in vitro and was again associated with worsened survival [24]. Studies in AML found a decreased expression of LSAMP in relapsed cases [20]. Prostatic adenocarcinoma studies linked LSAMP aberration with prostate cancer in AA patients, and with disease progression more frequently in AA patients [3,28]. Across disease stages, breakpoints in the LSAMP region have emerged among structural variation patterns, including in metastatic castration-resistant prostate cancers [4]. It has been suggested that LSAMP functions by downregulating RTKs and downstream oncogenic signaling [23]. There are various mechanisms of LSAMP downregulation in several cancers, by genomic rearrangements, most frequently deletions, epigenetic silencing, or potentially post-transcriptional or post-translational inactivation, each of which may be related to predisposition to disease progression. Current data unanimously support the notion that the downregulation of LSAMP is associated with oncogenesis in multiple cancer types, as well as worse outcomes for patients. Notably, the detection of LSAMP loss has potential to detect clinically significant malignancies. Additionally, the molecular mechanism by which LSAMP functions as a tumor suppressor gene by modulating known oncogenic pathways may provide new opportunities for treating patients with LSAMP associated malignancies.

**Figure 2:**
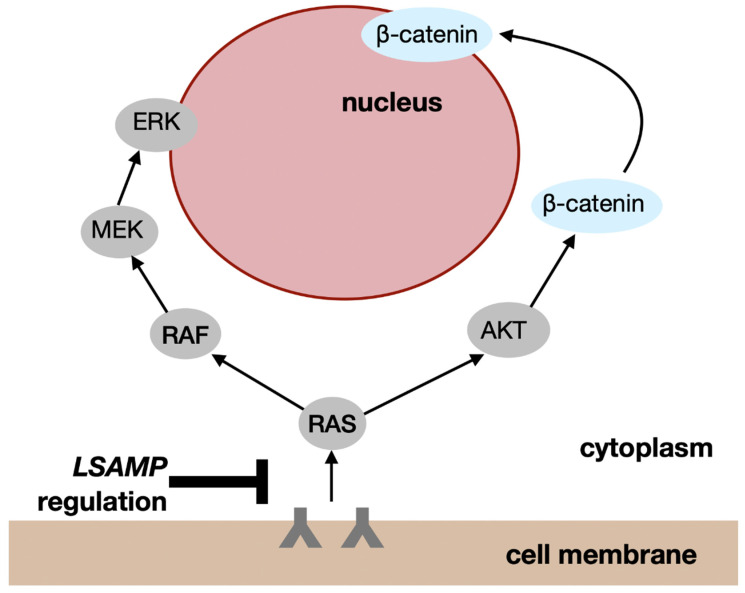
LSAMP losses in the setting of cancer may increase the expression of receptor tyrosine kinases on the surface of the cell, leading to the increased activation of tumorigenic ERK and Akt pathways and increased activation of β-catenin.

The goal of our review was to summarize published studies that investigate both the role of LSAMP deletion or decreased expression in tumorigenesis, and the clinical and biological changes associated with LSAMP gene defects (Table 2). Emerging data underscore that LSAMP losses in the setting of cancer can increase the expression of receptor tyrosine kinases on the surface of the cell, leading to the increased activation of tumorigenic ERK and Akt pathways and the increased activation of β-catenin (Figure 2). Akt is known to activate β-catenin via phosphorylation while deactivating GSK3, which inhibits the activity of β-catenin at baseline in the resting cell [40]. ERK is activated via the RAS/MAPK pathway, and then goes on to activate downstream pathways involved in cell proliferation, growth, and survival, including transcription factors and C-MYC as reported in AML [41]. The activation of the β-catenin pathway further promotes tumorigenesis via altered cellular metabolism, as evidenced in metabolomic data and emerging deutenomic data.

The mechanism of action of LSAMP is likely context dependent. The drosophila analog of IgLON, or Amalgam (Ama), was studied in a glial cell model and was found to function as a modulator of the receptor tyrosine kinase pathway [42]. However, Ama in glia was shown to indirectly increase receptor tyrosine kinase activity and downstream ERK signaling [42]. A knockdown of LSAMP in this context was subsequently performed, and in the same vein as Ama, increased expression of RTK pathway inhibitor SPROUTY2 was observed [42]. Effectively, the drosophila Ama, and LSAMP, worked in this model as an inhibitor of the inhibitor. Moreover, in astroglial cells, the expression of other members of the IgLON family increased fibroblast growth factor (FGF) signaling [42]. This contrasts with the currently understood action of LSAMP in other human adenocarcinoma models and the proposed suppressor role of LSAMP among other human malignancies, where LSAMP expression downregulates the RTK pathway and suppresses tumor growth. The differences between the mechanism of drosophila Ama versus human LSAMP suggests a context-dependent interaction with downstream RTK signaling.

A clinically important question that began to emerge from reports on LSAMP is the potential association between LSAMP deletion in malignancies and ancestry. A study conducted on analyses of 435 prostate cancer patients established a significant association between defects in the LSAMP gene and tumors of African ancestry [3]. Further studies of structural variations in prostate tumors identified LSAMP as a candidate driver gene and possible prognostic target for AA tumors [28]. In general, variations across ancestry have been established in some, but not all, types of cancer. In neuroblastoma, no association between ancestry and 5-year cause-specific survival was found in a study of 2119 pediatric neuroblastoma patients (age less than 18 years at diagnosis) [43]. The malignancies discussed in this review that have known associations with patient ancestry include renal cell carcinoma, lung adenocarcinoma, osteosarcoma, and AML. While Chen et al. did not stratify their results by ancestry, data show decreased survival rates in AA patients for every subtype of renal cell carcinoma [1]. Alaiwi et al. report that of 1829 patients with RCC who were provided germline genetic testing, pathogenic or likely pathogenic variants in the FH gene were more common in AA patients, while the CHEK2 gene was more common in non-African patients [44]. The study authors note that of the 1829 patient sample size, only 99 were of African descent, highlighting a need for larger studies of minority populations to lend more power to genetic associations [44].

Strong associations with ancestry can be seen in multiple genes implicated in lung adenocarcinoma. A study on the genomic sequencing data of 6238 lung adenocarcinoma patients included CA, AA, American Indian or Alaska Native (AI/AN), Asian, and other descents (Native Hawaiian/Pacific Islander, Hispanic/Latino, and interracial or multiracial) [45]. The distribution of ancestry in this study, as in RCC, encompassed mostly CA patients (84.89% CA, 8.64% Asian, 4.81% AA, 0.16% AI/AN, and 1.50% Other) [45]. The data demonstrated that point mutations in EGFR were three times higher in Asian patients than in others, and mutations in KRAS were significantly higher in CA, followed by AA, Other, AI/AN, and lastly Asian. The study did not evaluate for ancestry associations with LSAMP, while Chang et al. found decreased LSAMP expression in cell lines and tumor samples but did not correlate their findings with ancestry.

Studies on osteosarcoma, AML, and ovarian cancer have reported significant differences between ancestry but did not correlate findings with changes in LSAMP. A sample of 537 osteosarcoma tumors demonstrated a 15% higher incidence among AA patients and an 11% higher incidence among patients of Hispanic ancestry (HA) [46]. In a sample of 814 pediatric patients (age less than 21 years at diagnosis) with acute myeloid leukemia, AA and HA were more likely to have AML with t(8;21) [47]. Compared with CA and HA, AA patients had significantly higher risk of events, higher risk of death, and higher rates of early death within fifty days of diagnosis [47]. Among patients with CBF-AML, AA patients had the lowest event-free survival and the highest rates of early death [47]. Interestingly, the authors report that despite the favorable prognosis of CBF-AML, outcomes for African American patients with CBF-AML were similar to those of AML with higher risk cytogenetics [47]. In a study of 91,625 epithelial ovarian cancer patients, African American and Japanese American patients had a lower risk of developing cancer after adjusting for multiple variables [48]. The authors found no associations of endothelial ovarian cancer with age at menarche, obesity, smoking status, or diabetes among any ancestries [48]. However, the study only investigated associations with hormonal factors, and did not investigate genetic variations across ancestry. Genetic explanations for these findings in osteosarcoma, AML, and ovarian cancer remain unclear. In summary, discerning associations between ancestry and LSAMP-driven malignancies appears to be an exciting future direction.

## 11. Conclusions

The limitations of this review include variation in methodology across the included publications. The studies reviewed focused on different endpoints associated with LSAMP’s involvement in oncogenesis, including nodal metastasis, tumor stage, tumor aggressiveness, or rates of cell line growth. Additionally, differences by ancestry have been found in most cancers, but studies vary in investigating correlations between genomic changes and ancestry. Cancers such as RCC, prostatic adenocarcinoma, lung adenocarcinoma, and AML have defined genetic risk factors for the disease, and cancers such as RCC, prostatic adenocarcinoma, osteosarcoma, AML, and ovarian have significant differences in disease outcome between ancestries. RCC, prostatic adenocarcinoma, AML, and ovarian cancer have stratified genetic risk factors by ancestry, but other cancers have not investigated the role of LSAMP or other significant factors in the context of the ancestries of patients. Clinical applications of LSAMP’s function to diagnostics, prognostics, and therapies will require continued investigation. Further research needs to be carried out to determine how integrin signaling contributes to adherence in the presence and absence of LSAMP. The broader implications of these findings include the development of treatment therapies targeted to tumors harboring defective LSAMP that will allow for more the informed risk- and perhaps therapeutic stratification of patients.

## 12. Future Directions

The clinical application of LSAMP has potential in diagnostics, prognostics, risk stratification, and the development of treatment therapies. Given the association of LSAMP aberration with malignancy, and of signal transduction pathways with tumorigenesis, continued studies will be needed to assess their involvement in altered cellular genetics and the metabolome. Further research will determine how LSAMP influences cell adherence in the presence of RTK and integrin signaling. Additionally, investigating associations between ancestry and LSAMP-driven malignancies appears to be an exciting future direction.

## Figures and Tables

**Table 1:** Studies investigating LSAMP in malignancies.

Study Objectives	Study Methods	Authors	Cancer Type	Findings
Investigate t(1;3) breakpoint-spanning gene LSAMP	FISH, Southern blot, Northern blot, expression plasmids	Chen et al., 2003 [18]	RCC	LSAMP associated with chromosome 3 translocations implicated in hereditary RCC LSAMP expression decreased with LSAMP promotor methylation LSAMP expression increased with methylation inhibitor LOH of LSAMP locus associated with tumor development independent of methylation Cancer cells did not grow following LSAMP transfection, controlled for apoptosis
Determine IgLON expression in tumor samples	RT-PCR	Ntougkos et al., 2005 [19]	Ovarian	Reduced copy number, LSAMP expression in tumor samples LSAMP alterations were a prognostic factor and associated with decreased overall survival
Identify LSAMP by array comparative genomic hybridization	Array CGH, FISH, RT-PCR	Kresse et al., 2009 [14]	Osteosarcoma	LSAMP expression decreased with LSAMP promotor methylation Decreased LSAMP expression associated with decreased survival
Investigate recurrent focal CN Changes and LOH at Chromosome 3q13.31	CN analysis, RT-qPCR, FISH	Pasic et al., 2010 [15]	Osteosarcoma	Cancer cell growth increased with decreased LSAMP expression Suppression of LSAMP increased transcription of cyclins
Genomic profiling of CBF-AML	SNP array, CN-LOH analysis, FISH	Kühn et al., 2012 [20]	AML	LSAMP expression decreased in bone marrow samples of relapsed AML
Investigate LSAMP expression in tumor growth	qPCR, Western blot, CN assay, immunofluorescence confocal microscopy, flow cytometry	Barøy et al., 2014 [21]	Osteosarcoma	Reduced copy number, LSAMP expression in tumor samples Cancer cell growth increased with decreased LSAMP expression LSAMP addition delayed tumor growth in vivo
Investigate association of LSAMP with aggressive AA prostate cancer	WGS, SNP array, FISH analysis	Petrovics et al., 2015 [3]	Prostate	LSAMP expression decreased or undetectable in cancer cell lines Loss of LSAMP associated with recurrence, was a prognostic factor
Identify recurrent focal CNVs and putative targeted driver genes	CNV analysis	Zhang et al., 2016 [22]	Ovarian	Reduced LSAMP expression in tumor samples
Investigate role of LSAMP in tumor growth, RTK expression, and EMT mechanism of metastasis	FISH analysis, RT-qPCR, Western blot, IF assay, TOP/FOP Flash assays, ECM-cell adhesion assays, cell-cell adhesion assays, MTT assays	Babcock et al., 2019 [23]	Prostate	Cancer cell growth decreased following LSAMP transfection LSAMP downregulated RTKs, blocked ERK, Akt pathways LSAMP loss increased beta-catenin activity Loss of LSAMP caused reversion to mesenchymal cell-cell adhesion characteristics LSAMP expression reduced tumor growth in vivo
Investigate LSAMP downregulation and survival prognosis	NGS, shRNA knockdown	Chang et al., 2021 [6]	Lung	Reduced LSAMP expression in tumor samples Increased nodal metastasis associated with decreased LSAMP More advanced tumor stage associated with decreased LSAMP Silencing LSAMP decreased epithelial markers, increased mesenchymal markers in cancer cells
Identify LSAMP as tumor suppressor gene	WGS, SNP array, Sanger sequencing analysis, shRNA knockdown	Martinez-Monleon et al., 2023 [24]	Neuroblastoma	Cancer cell growth increased with LSAMP silencing Cancer cell growth decreased following LSAMP transfection Decreased LSAMP expression associated with decreased event-free and overall survival

**Table 2:** Changes associated with the LSAMP gene in cancers.

Type of Cancer	Type of LSAMP Change
Clear cell renal cell carcinoma	Methylation
Prostatic adenocarcinoma	Deletion, duplication, methylation
Lung adenocarcinoma	Methylation
Osteosarcoma	Deletion, methylation
Neuroblastoma	Deletion, duplication, translocation
CBF-AML	Deletion
Ovarian epithelial	Deletion
